# Emerging panorama of functional near-infrared spectroscopy in Latin America

**DOI:** 10.1117/1.NPh.13.S1.S13002

**Published:** 2025-09-12

**Authors:** Edgar Guevara, Rickson C. Mesquita, Felipe Orihuela-Espina

**Affiliations:** a Universidad Autonoma de San Luis Potosí, Faculty of Science, San Luis Potosí, Mexico; b Universidad Autonoma de San Luis Potosí, CIACYT, San Luis Potosí, Mexico; c University of Birmingham, School of Computer Science, Birmingham, United Kingdom; d University of Campinas, Institute of Physics, Campinas, Brazil

## Abstract

Neurophotonics Associate Editors Edgar Guevara and Rickson C. Mesquita, along with Felipe Orihuela-Espina, share their perceptions of the evolution of functional near-infrared spectroscopy in Latin America based on a retrospective literature review and the narratives of three other scientists from the region.

## 1 Introduction

Over the past two decades, functional near-infrared spectroscopy (fNIRS) has gained steady traction among researchers across Latin America. This growing interest reflects both the promise of fNIRS as a neuroimaging tool and the complex realities of advancing research within diverse and often resource-limited regional contexts. Since the first Latin American researchers began engaging with fNIRS, the technique has expanded across multiple countries in the region, achieving greater autonomy in data acquisition and analysis, and making meaningful contributions to the global fNIRS community.

A literature search in Clarivate’s Web of Science using the keywords “fNIRS,” “functional near-infrared spectroscopy,” “diffuse optics,” or “diffuse optical imaging” in titles or abstracts, and “brain” or “cerebral” in any part of the text, filtered by institutional affiliations from any Latin American country, provides valuable insights into the development of fNIRS in the region. This quantitative information can be further enriched by the lived experiences of six researchers who have advanced fNIRS in the area. Their perspectives highlight not only the scientific potential of the technique but also the unique challenges and opportunities of conducting research in Latin America.

Together, this combined approach provides a comprehensive and holistic view of fNIRS development in the region, clearly demonstrating the potential of fNIRS as a valuable tool for addressing regionally relevant questions in neuroscience.

## 2 Beginnings

Although the idea of using near-infrared light to noninvasively monitor cerebral hemodynamics dates back to the seminal work of Jöbsis in the late 1970s,^1^ it was not until key advances in optical instrumentation from the mid-1980s and early 1990s that near-infrared spectroscopy (NIRS) techniques gained wider scientific traction. Early research focused primarily on demonstrating the feasibility of the approach and understanding how optical signals reflected physiological and hemodynamic changes associated with brain activity or brain injuries.^2^ By the mid-1990s, efforts began to explore the relationship between functional NIRS (fNIRS) signals and brain activity more in-depth, including correlations with BOLD-fMRI, which was also being developed during this time.

The earliest fNIRS studies involving researchers from Latin America emerged right after this feasibility period, in the early 2000s, primarily driven by international collaboration and personnel exchange. Partnerships with institutions in North America and Europe played a key role in facilitating knowledge exchange, providing training, and granting access to fNIRS technologies for Latin American researchers. For this reason, most of the initial efforts in fNIRS research in Latin America reflected the state of fNIRS development in the Northern Hemisphere.

**Fig. 1:**
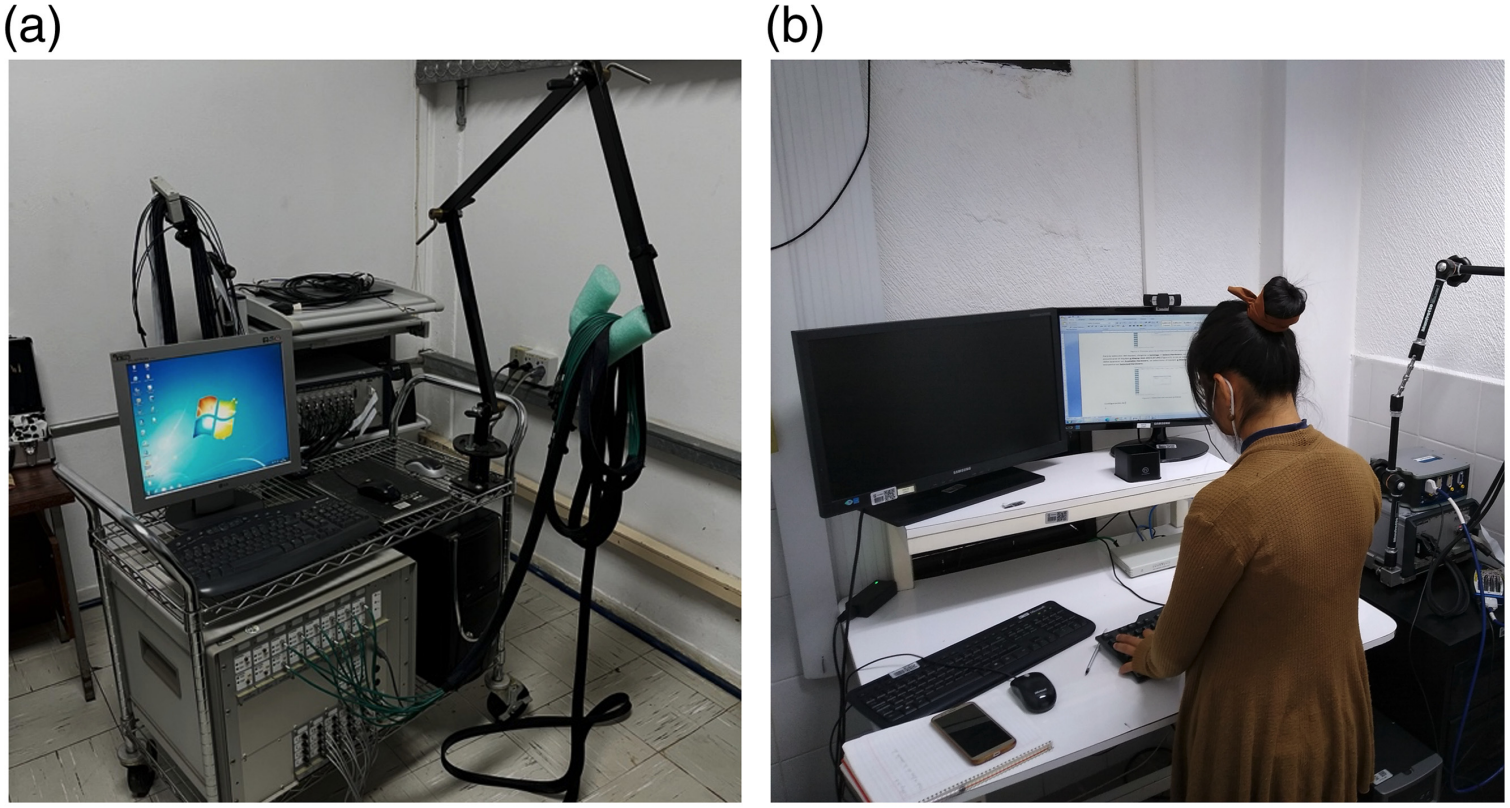
Two of the early fNIRS systems available in Latin America. (a) CW6 (TechEn, Milford, USA), the first NIRS system acquired in Brazil in 2008. The device remains functional at the University of Campinas and comprises a multichannel system (20 detectors, 10 sources with two wavelengths each) for continuous-wave NIRS functional experiments. (b) NIRScout (NIRx Medical Technologies, Berlin, Germany), an fNIRS system available at INAOE.

Early contributions co-authored by researchers from Brazil date back to 2004 and focused on methodological advances, such as estimating the cerebral metabolic rate of oxygen,^3^^,^^4^ investigating neurovascular coupling,^5^ studying resting-state functional connectivity,^6^ and improving fNIRS data analysis methods.^7^ These projects were mostly conducted in collaboration with research groups in the USA and Canada, with Latin American researchers centered at the University of Campinas (UNICAMP) in Brazil, led by Roberto Covolan and Rickson Mesquita, who at the time was a PhD student and would go on to defend the first doctoral thesis on fNIRS in Brazil in 2009.^8^ These pioneering efforts were made possible by financial support from the São Paulo Research Foundation (FAPESP) – one of the first large-scale, long-term collaborative neuroscience funding initiatives in Brazil.^9^ This funding also enabled the acquisition of Brazil’s first commercial multichannel continuous-wave NIRS device in 2008, produced by TechEn (Fig. 1), which motivated local experimental protocols with fNIRS targeting applied and clinical neuroscience in the 2010s.

Independent fNIRS applications in functional neuroimaging also began to emerge in Latin America in the early 2000s, primarily through collaborations with European institutions. Some of the earliest studies, dating back to 2003, focused on cognitive function in neonates and involved researchers from Chile and Argentina.^10^^–^^12^ Argentinean researchers further contributed to investigations of motor imagery^13^^,^^14^ and conducted some of the first studies with fNIRS exploring social interactions in teacher-student duos.^15^ In Mexico, the Universidad Nacional Autónoma de México (UNAM) acquired a commercial fNIRS system from Hitachi by 2010, laying the groundwork for early local applications. At the same time, researchers in the south of Brazil began using fNIRS to investigate language processing^16^^,^^17^ and emotional responses.^18^^,^^19^ By the mid-2010s, published contributions from other parts of the region, including Colombia^20^ and several parts of Mexico,^21^^–^^24^ began to appear. Likely, fNIRS use was already more widespread than these publications suggest, with many early efforts limited to conference presentations or unpublished work – indicating a broader engagement with fNIRS that can be captured through our bibliographic search alone.

## 3 Consolidation and Establishment of fNIRS-Focused Research

**Fig. 2:**
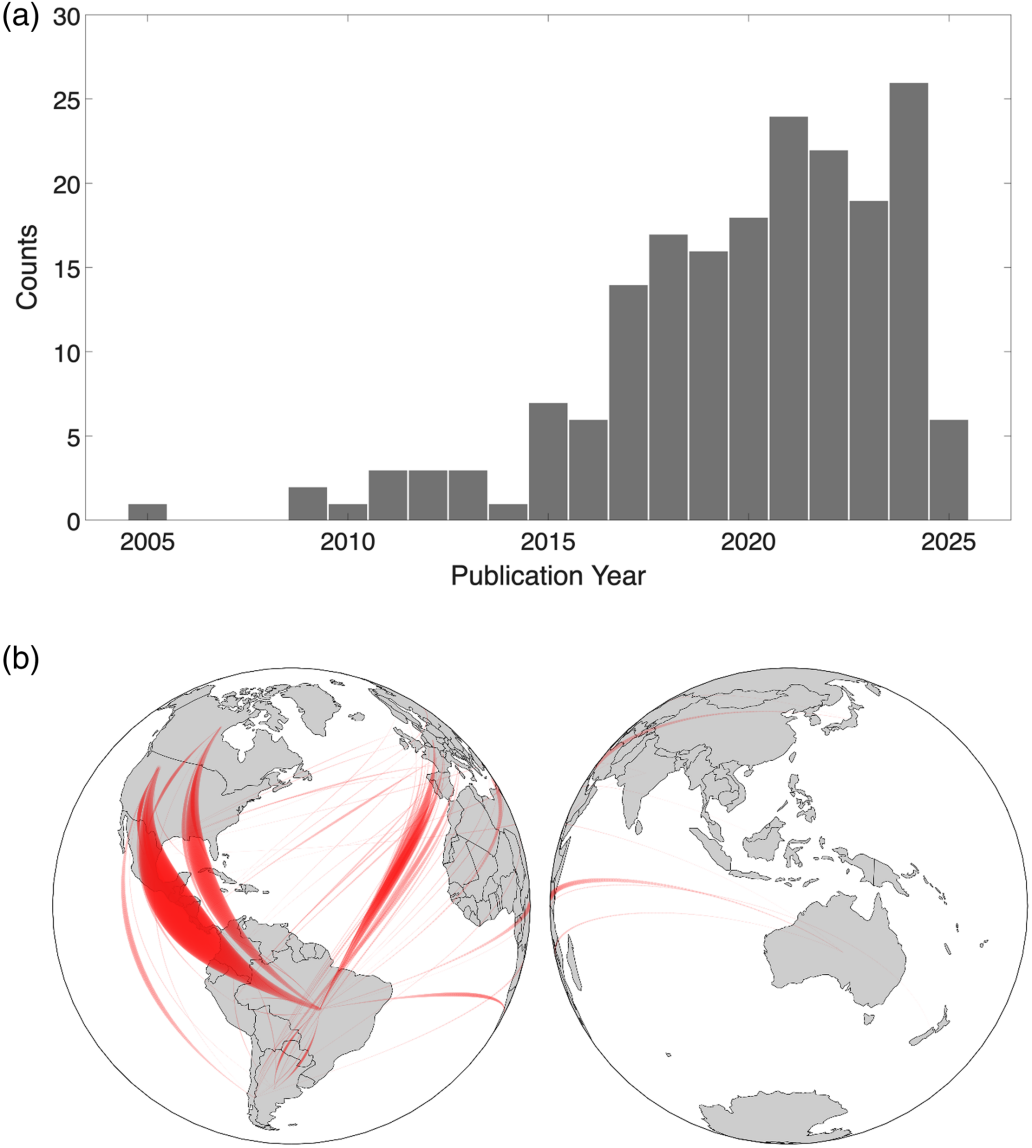
(a) Number of yearly publications in fNIRS with the participation of researchers from Latin American affiliations. (b) Collaboration network between fNIRS researchers from Latin America and other parts of the world. Database source: Clarivate’s Web of Science.

Building on the foundation laid by early international collaborations – often based on data collected abroad – the adoption of fNIRS in Latin America has grown significantly over the past decade (Fig. 2). This expansion mirrors global trends in fNIRS research worldwide but has been further accelerated by the consolidation of local research groups and the recognition of neuroscience into the regional research agenda.

Motivations for adopting fNIRS in Latin America are both practical and scientific. Scientifically, fNIRS techniques are versatile across spatial and temporal scales, which makes them well-suited to address neuroscience questions across the lifespan and in ecologically valid experimental settings. Practically, fNIRS offers a more accessible and affordable alternative for low-resource contexts, helping to decentralize neuroscience research from major local research institutions and enabling participation from institutions in more remote or underserved areas of the region.

Its portability, rapid setup, and lower sensitivity to motion artifacts compared to modalities like fMRI or EEG make fNIRS particularly suitable for studying infants and young children. In Brazil, for example, the Technology Centre in Molecular Medicine at the Federal University of Minas Gerais (UFMG) has employed fNIRS since 2010 to study brain development in premature infants^25^^–^^27^ and in the context of congenital infectious diseases.^28^ fNIRS was also employed to study microcephaly caused by congenital Zika virus infection.^29^ Similar efforts have emerged in Mexico, where researchers have established programs to study balance and posture,^30^ as well as neurodegenerative diseases such as Parkinson’s disease using portable fNIRS devices in the local population of San Luis Potosí.^31^^–^^33^ More recently, researchers from Peru worked with collaborators to combine fNIRS with other modalities to study newborn brain activity.^34^ These examples highlight the role that fNIRS has played in developing a framework centered around Responsible Research and Innovation (RRI) in Latin America, directly addressing local public health priorities that are often underrepresented in research agendas of high-income countries.

As fNIRS research became more established, several Latin American institutions began attracting international researchers—especially from other Latin American countries, Africa, Europe, and the Middle East—who have contributed to the growth of regional expertise. At the Mackenzie Centre for Research in Childhood and Adolescence in São Paulo, researchers launched a program focused on the neural correlates of social touch, relevant for understanding early childhood socioemotional development. Similar research programs have been developed at the National Institute of Astrophysics, Optics and Electronics (INAOE) in Puebla, Mexico. At the Federal University of ABC (UFABC), fNIRS became a central tool for research programs in applied neuroscience and studies of brain function in real-world contexts. This includes studies using fNIRS in naturalistic settings,^35^^–^^37^ neuroscience and music,^38^^–^^40^ as well as the intersection of neuroscience and education.^41^^–^^44^ These studies not only contribute to global scientific knowledge but also offer practical insights that can inform interventions relevant to the well-being and development of Latin American populations.

In parallel, there has been a notable rise in methodological and technological innovation related to fNIRS in the region. While sparse, continuous-wave NIRS remains the most widely used modality, alternative technologies are gaining traction. One example is a portable, high-density device for diffuse optical tomography, specifically designed to address malnutrition in Colombia in collaboration with researchers in the USA.^45^ In Brazil, commercial frequency-domain diffuse optical spectroscopy (FD-DOS) systems have been utilized to conduct longitudinal studies during exercise and to more accurately quantify cerebral hemodynamics in local populations with darker skin and specific hair characteristics—physiological features more often underrepresented in the literature.^46^^–^^48^ Brazil has also begun developing NIRS-based technology in the region, as evidenced by its first diffuse correlation spectroscopy (DCS) device for cerebral blood flow measurement in 2015.^49^ This system has since been applied in clinical research involving neurocritical care patients at the Clinical Hospital of UNICAMP.^50^^,^^51^ In Mexico, a multimodal EEG-fNIRS platform using a Raspberry Pi 4 was built for deployment in rural communities.^52^ In parallel, clinical researchers from Colombia validated fNIRS for monitoring cerebral autoregulation in comatose patients.^53^ Such efforts highlight the growing regional technical expertise and the promise of DCS and other diffuse optical technologies for low-cost, continuous bedside monitoring in low-resource clinical environments.

Methodological development has also matured with technological advancements, driven by the need for improved signal processing and data analysis in fNIRS. This includes studies on depth sensitivity in time-resolved reflectance measurements involving researchers from Argentina,^54^ new processing techniques in Brazil to enhance signal reliability,^55^^–^^57^ novel signal processing for multimodal fNIRS in Cuba,^58^ and intelligent data analysis and image interpretation developed Mexico.^59^^–^^61^ Two widely adopted tools originating from the region include a protocol for resting-state analysis using graph theory^62^ and an open-source toolbox for probe arrangement guided by brain regions of interest.^63^ Both contributions show how methodological innovations from Latin America are helping shape fNIRS research globally.

As expertise has grown, so too has integration between groups developing fNIRS methods and those applying them to scientific and clinical questions. This has led to a more resilient and interconnected fNIRS community in Latin America, with the participation of fNIRS companies. As a result, there are now more training opportunities for early-career researchers and an increase in collaborative projects within the region, which in turn have strengthened funding prospects and improved regional dissemination of research outputs.

Several initiatives have played a key role in supporting this consolidation. Since 2013, the Brazilian Institute of Neuroscience and Neurotechnology (BRAINN)—another long-term initiative supported by FAPESP to develop neuroscience in São Paulo—has served as a national and regional hub, training researchers from across Latin America and supporting the integration of fNIRS with other neuroimaging modalities. More recently, the Brazilian government established the National Institute for Responsible Neurotechnology (NeuroTec-R) in Minas Gerais, a multidisciplinary network that includes researchers from Latin America and beyond. The Santos Dumont Institute in Northeast Brazil, a social organization dedicated to neuroscience and neuroengineering, has also applied fNIRS in various areas, including maternal and child health, as well as the rehabilitation of people with disabilities. Private institutions such as the Hospital Israelita Albert Einstein have also played an important role in translating fNIRS into clinical research.^64^^–^^67^

The consolidation of the Latin American fNIRS community has been accompanied by the expansion of training opportunities and the organization of local events. Since 2017, BRAINN has hosted several in-person, hands-on training courses, supporting the development of early-career researchers across multiple institutions around Brazil and other South American countries. In 2019, two international schools on Biophotonics were held—one at the University of São Paulo in Brazil^68^ and another at UNAM in Mexico.^69^ In parallel, private sector initiatives by NIRx Medical Technologies and Brain Support have also delivered educational workshops and training sessions across different regions of Latin America.^70^^,^^71^ The first Mexican Symposium on Near-Infrared Spectroscopy Neuroimaging (MexNIRS) was held in 2017,^72^ bringing together research groups from across the region and abroad. A major milestone in community-building was the first Latin American NIRS Meeting (LatAm NIRS), held virtually in December 2022. The event was conducted entirely in Spanish and Portuguese and brought together 134 researchers from Latin America, Portugal, and Spain. These initiatives reflect a growing regional identity and a shared commitment to advancing fNIRS research, and they represent important progress toward a more inclusive and collaborative scientific ecosystem.

## 4 Regional Challenges in fNIRS Research

Despite the notable scientific progress and some clear successes, the broader reception of fNIRS research from Latin America remains mixed. It is not uncommon for studies conducted exclusively within the region to face unjustified skepticism during peer review, with concerns about data quality or methodological rigor sometimes rooted more in prior assumptions than in the evidence presented. Perceived language and cultural barriers, frequently overstated or unfounded, also contribute to this bias. These challenges prompt the need to broaden our understanding of scientific merit beyond standards historically shaped by specific cultural contexts. Embracing diversity in scientific thought—while keeping rigorous methodological standards that are not culturally bound—is essential to move beyond superficial commitments to equity and toward genuine inclusion.

Beyond these social and systemic challenges, researchers in Latin America also face practical and logistical barriers. The geographic isolation of South American countries increases transportation costs and limits access to fNIRS equipment, scientific events, and wide specialized training. Scientific research itself is still relatively young in many parts of the region, both in historical development and in how it is prioritized at the national level (often reflected in the low proportion of GDP allocated to research and innovation). In some cases, research budgets have been completely suspended for entire fiscal years. This lack of stable, long-term science policy makes it difficult to sustain a consistent research agenda. Ongoing sociopolitical instability in the region further complicates the development of strong, resilient research infrastructures and long-term institutional support.

It is also important to acknowledge the substantial disparities that exist within Latin America. Countries in the region differ widely in socioeconomic conditions and the degree to which research is prioritized, resulting in uneven access to resources and opportunities. Most of the examples cited above come from Brazil and Mexico—the two largest economies in Latin America. In smaller or less economically developed countries, maintaining a stable research program over the long term presents even greater challenges. Addressing this intra-regional inequality and pursuing greater equity in research capacity and collaboration remains a key long-term objective.

To navigate these challenges, international exposure through collaborations has played a role in sustaining Latin American fNIRS research and reinforcing its credibility. These partnerships have increased visibility and brought tangible benefits to both sides, including access to broader funding streams, enhanced experimental paradigms and participant demographics, and valuable opportunities for researcher exchange and development.

One persistent limitation is the lack of a well-established technological and industrial base to support fNIRS research. Most fNIRS equipment or optical parts must be imported, subjecting researchers to complex customs bureaucracy, high import tariffs, and long delays. These challenges are compounded by economic instability; unfavorable currency exchange rates mean that fNIRS systems can cost up to four times more than in high-income countries (in a flat rate comparison), placing a disproportionate financial burden on Latin American institutions.

Human resources also remain a critical bottleneck. Although regional training initiatives and the return of researchers from abroad have increased local capacity, the absence of long-term research funding policies makes scientific careers unstable. This, in turn, drives early- and mid-career researchers to seek more stable positions elsewhere, contributing to a chronic shortage of local technical expertise and increasing demand for training. Recent efforts by the Society for functional Near-Infrared Spectroscopy (SfNIRS), such as offering remote training in Spanish, represent necessary steps toward reducing these disparities. However, hands-on experience—especially for application-focused research—is essential and remains difficult to assess.

Improving research dissemination represents another persistent challenge. Hosting major conferences in Latin America could help reduce geographic and economic barriers to participation while increasing local visibility. Currently, only the most stable research groups at major centers can afford in-person attendance at international conferences. While virtual options offer some relief, they still restrict participation due to time zone differences and limited networking opportunities.

On the other hand, fNIRS research in Latin America brings unique strengths that help counterbalance these obstacles. For instance, a friendly and welcoming research culture contributes to high participant recruitment rates in fNIRS research studies—often exceeding 75%. In some countries, legal restrictions on participant compensation further reduce study costs and enhance feasibility. While access to higher education is still unequal, top Universities in the region produce highly skilled, pragmatic, and resilient students with strong scientific training—attributes that help Latin American researchers stand out internationally. These distinctive features provide a solid foundation for continued growth in the field.

## 5 Conclusions

Looking ahead, lessons from the past two decades offer a clear path forward. Building strong local and international partnerships, staying connected to the global scientific community, and investing in training and capacity-building initiatives will be essential for sustaining progress. Despite persistent limitations in infrastructure, funding, and geography, the outlook for fNIRS research in Latin America is more promising today than it was 25 years ago.

## Data Availability

The code and data used to create Fig. 2 is openly available at https://github.com/guevaracodina/fNIRSlatAm.
